# The Neuroprotective Role of Melatonin in Intracerebral Hemorrhage: Lessons from an Observational Study

**DOI:** 10.3390/jcm14051729

**Published:** 2025-03-04

**Authors:** Maria Ioanna Stefanou, Katharina Feil, Sofiya Zinsser, Vasileios Siokas, Sara Roesch, Jennifer Sartor-Pfeiffer, Kornelia Laichinger, Constanze Single, Sophia Kindzierski, Benjamin Bender, Ulf Ziemann, Annerose Mengel

**Affiliations:** 1 Department of Neurology & Stroke, Eberhard-Karls University of Tübingen, 72076 Tübingen, Germany; 2 Hertie Institute for Clinical Brain Research, Eberhard-Karls University of Tübingen, 72076 Tübingen, Germany; 3 Department of Thoracic, Cardiac and Vascular Surgery, Eberhard-Karls University of Tübingen, 72076 Tübingen, Germany; 4 Department of Neurology, University Hospital of Larissa, Faculty of Medicine, School of Health Sciences, University of Thessaly, 41100 Larissa, Greece; 5 Department of Neuroradiology, Eberhard-Karls University of Tübingen, 72076 Tübingen, Germany

**Keywords:** intracerebral hemorrhage, melatonin, neurological outcome, disability, mortality

## Abstract

**Background/Objectives:** Growing evidence is underscoring the neuroprotective properties of melatonin, particularly its anti-inflammatory, anti-apoptotic, and antioxidant effects. Preliminary findings suggest that it has the potential to attenuate secondary brain injury following intracerebral hemorrhage (ICH). This observational study aimed to investigate the effect of melatonin on post-ICH mortality and functional outcomes. **Methods:** We conducted an exploratory analysis of data from a single-center, non-randomized, prospective cohort study involving 177 non-ventilated patients with spontaneous ICH consecutively admitted to the Stroke Unit at the University Hospital of Tübingen, Germany, between December 2015 and December 2020. Patients received either the best standard of care (control group) or the best standard of care plus melatonin (2 mg nightly), initiated within 24 h of symptom onset and continued until discharge. The primary endpoint was mortality at discharge, while secondary endpoints included mortality at 90 days and favorable outcomes (modified Rankin Scale [mRS] ≤ 2) at both discharge and a 90-day follow-up. To minimize baseline differences, propensity score matching (PSM) was employed in the secondary analysis. Additionally, ordinal mRS shift analysis was performed to assess the patients’ functional status at discharge. **Results:** In the full cohort (84 melatonin-treated patients vs. 93 controls), melatonin was not associated with any of the primary or secondary outcomes. In the PSM cohort (38 melatonin-treated patients vs. 38 controls), mortality at discharge was three times lower in the melatonin group compared to the control group (2.6% vs. 7.9%), although this trend did not reach statistical significance (ORadj: 0.372; 95% CI: 0.036–3.843; *p* = 0.407). Ordinal mRS analysis revealed no significant association between melatonin and functional status at discharge (common OR: 0.762; 95% CI: 0.327–1.773; *p* = 0.527). Similarly, the melatonin treatment was not associated with 90-day mortality (ORadj: 1.519; 95% CI: 0.295–7.826; *p* = 0.617) or the functional outcome at 90 days (ORadj: 0.626; 95% CI: 0.198–1.983; *p* = 0.426). **Conclusions:** Although 2 mg of melatonin daily did not significantly reduce mortality or improve functional outcomes in ICH patients, robust preclinical evidence and the favorable safety profile of melatonin warrant its further exploration in adequately powered, randomized-controlled clinical trials to evaluate optimized dosing regimens.

## 1. Introduction

Intracerebral hemorrhage (ICH) accounts for 10–25% of all strokes, with an age-standardized incidence of 41.8 per 100,000 person-years [1,2]. Associated with a nearly fourfold higher mortality risk compared to acute ischemic stroke (AIS), ICH contributes substantially to the global disease burden, resulting in over 60 million disability-adjusted life years (DALYs) annually [1,3]. Long-term outcomes for ICH remain poor, with approximately half of patients surviving at one year, and only 50% of survivors achieving functional independence [4].

The therapeutic options for ICH have historically been limited to early neurosurgical interventions, blood pressure management, anticoagulant reversal, and the treatment of ICH-related complications [5,6,7,8,9,10]. Recent advances [6], such as acute care bundles (INTERACT3) [11], intensive ambulance-delivered blood-pressure lowering (INTERACT4) [12], the use of andexanet alfa for reversing factor Xa inhibitor anticoagulation (ANNEXa-I) [13], early minimally invasive hematoma evacuation (ENRICH) [14], and decompressive hemicraniectomy for large deep ICH (SWITCH) [15], have improved outcomes. However, effective neuroprotective strategies to prevent neuronal injury, driven by mechanisms such as oxidative stress, inflammation, and excitotoxicity, remain an unmet need [7]. Secondary brain injury after ICH is mediated by cerebral edema, neuroinflammation, and biochemical toxicity from blood products, leading to mitochondrial dysfunction, apoptosis, and neuronal damage [16,17,18].

Melatonin, a lipid-soluble hormone synthesized primarily by the pineal gland and know for regulating circadian rhythms, has recently garnered interest for its multifaceted neuroprotective properties, particularly in the context of ICH [19]. Preclinical studies using ICH models suggest that melatonin can reduce neuroinflammation, oxidative stress, cerebral edema, and blood–brain barrier disruption while mitigating apoptosis [20,21]. Clinical evidence, though limited, also suggests these benefits. In a randomized, non-controlled, double-blind study of 40 intubated adult patients with ICH, the administration of 30 mg of melatonin daily within 24 h of symptom onset significantly shortened the duration of their ICU stay and marginally reduced the duration of mechanical ventilation [22]. Although the reduction in mortality was not statistically significant, the treated group exhibited a mortality rate that was half that of the control group. Similarly, in a double-blind randomized controlled clinical trial (RCT) involving 52 patients with traumatic ICH, a regimen of 3 mg of melatonin daily decreased reliance on sedatives and shortened ventilation times [23]. Furthermore, there is limited evidence suggesting cognitive improvement in a delirious ICH patient treated with 8 mg of the melatonin agonist ramelteon, supporting melatonin’s potential in combating post-ICH neurocognitive deficits [24].

These findings underscore the need for larger-scale studies to evaluate the potential neuroprotective efficacy of melatonin in ICH. In this study, we investigated whether melatonin administration is associated with reduced mortality and improved functional outcomes at discharge and at a 90-day follow-up.

## 2. Materials and Methods

### 2.1. Study Design and Regulations

This study is an exploratory analysis of a single-center, non-randomized, prospective cohort study evaluating melatonin administration in ICH patients. The study includes a subset of data from a previously published cohort, the design and details of which have been extensively described elsewhere [25]. Consecutive patients with ICH were included and either treated with melatonin for post-stroke delirium (PSD) prevention or received the best medical care without melatonin (control group). Individual informed consent from the participants was waived, as clinic-wide consent has been implemented for the use of de-identified routine treatment data for research purposes. The protocol for the present study (protocol number 752/2018BO2) was approved by our Institutional Ethics Committee.

### 2.2. Study Population and Exclusion Criteria

We enrolled consecutive adult patients diagnosed with spontaneous intracerebral hemorrhage (ICH) (according to the International Classification of Diseases, 10th Revision [ICD-10] code I61) who were admitted to the intensive care unit (ICU) and/or Stroke Unit (SU) of the University Hospital of Tübingen between 1 December 2015 and 31 December 2020. The following exclusion criteria were applied: (i) an ICU/SU stay duration of <24 h; (ii) a diagnosis of subdural/epidural hemorrhage, hemorrhage from a co-localized tumor (non-stroke), subarachnoid hemorrhage, ischemic stroke with hemorrhagic transformation (stroke, non-ICH), or ICH due to structural lesions based on the SMASH-U classification system for ICH [26]; (iii) patients on mechanical ventilation; (iv) patients who underwent palliative care; and (v) patients who underwent neurosurgical intervention directly following their baseline CT scan.

### 2.3. Melatonin Administration

Patients in the melatonin-treated cohort received melatonin supplementation within the first 24 h of ICH onset (a single dose of 2 mg/day at 8 p.m.), following the standard operating procedure (SOP) for post-stroke delirium (PSD) prevention, as previously described [16], until ICU/SU discharge. The rationale for this SOP included the limited therapeutic options for PSD prevention, melatonin’s well-established safety profile, and emerging evidence of its neuroprotective effects in stroke and critically ill patients [25,27,28,29]. The 2 mg dose was selected based on prior data suggesting its efficacy in stroke-related PSD prevention and its established safety profile in this population.

### 2.4. Data Collection

Patient data, including demographic information, medical history, neuroimaging findings, in-hospital clinical parameters, and mortality during hospitalization, were extracted from the clinical information system (Intellispace Critical Care and Anesthesia information system, Philips Healthcare, Amsterdam, The Netherlands) of the studied ICU/SU. An attending vascular neurologist (KF) blinded to the baseline clinical, imaging, and demographic characteristics and in-hospital management of these patients, assessed their mRS at discharge. Another attending vascular neurologist (AM) blinded to the baseline clinical, imaging, and demographic characteristics and in-hospital management of the patients collected data regarding their functional outcome at 90 days after the index event via a structured telephone interview [25].

### 2.5. Outcomes

The primary outcome was mortality at discharge. Secondary outcomes included mortality at 90 days and favorable outcomes at discharge and at the 90-day follow-up. A favorable outcome was defined as an mRS of 0–2 [30].

### 2.6. Statistical Analysis

The normality of the data was assessed using the Shapiro–Wilk test. For categorical variables, Pearson’s chi-squared tests were calculated for between-group differences. For continuous variables, we used Mann–Whitney U tests, due to the observed non-normal distribution of the data. Values are presented as total numbers (*n*), with their respective percentages (%), and as medians, with their respective interquartile ranges (IQR), for categorical and continuous variables, respectively.

For the primary analyses of all primary and secondary outcomes of interest, a binary logistic analysis was performed, adjusting for variables with significant intergroup differences to control for confounding. In our secondary analyses, baseline differences in clinical covariates between patients treated with melatonin and controls were balanced using propensity score (PS) matching [5]. Propensity scores were calculated for all baseline parameters presenting intergroup differences (*p* < 0.05). Treated patients and controls were matched 1:1 using nearest neighbor matching, with a matching tolerance of 0.2. Standardized differences were estimated to compare their baseline characteristics before and after PSM, with imbalance being defined as an absolute value greater than 0.10 (a small effect size). Binary logistic regression analysis was performed using the PS-matched cohort while further controlled for residual between-group differences for the primary and secondary outcomes of interest. In addition, analysis of the secondary outcome, the modified Rankin scale score at discharge, was performed with the use of ordinal logistic regression, after confirmation that the proportional odds assumption was not violated, and adjusted for the variables that significantly differed between the groups after PSM. Secondary analyses were also performed with favorable outcome defined as an mRS of 0–3. A two-sided *p* < 0.05 was considered significant. All analyses were performed using SPSS 29 (IBM, Armonk, NY, USA).

## 3. Results

### 3.1. Patient Characteristics

Out of the 339 ICH patients included in our previously published cohort [25], 162 patients were excluded (*n* = 16 due to an SU stay duration of <24 h; *n* = 51 due to SMASH-U classification [26]; *n* = 78 due to mechanical ventilation; *n* = 8 due to palliative care; and *n* = 9 who underwent direct neurosurgical intervention). The remaining 177 patients (mean age [SD]: 71 (15) years, 46.3% female) that met the inclusion criteria for the present analysis comprised 84 patients (mean age [SD]: 75 (13) years, 45.2% female) in the melatonin treatment group and 93 (mean age [SD]: 68 (16) years, 47.3% female) in the control group. Their baseline characteristics are summarized in Table 1.

Significant between-group differences were observed for age, hypercholesterolemia, and prior treatment with direct oral anticoagulants (DOACs). After PSM, 38 ICH patients treated with melatonin (mean age [SD]: 76 (11) years; 42.1% female; median NIHSS on admission [IQR]: 5 (1, 9)) were compared to 38 ICH patients without melatonin (mean age [SD]: 76 (11) years; 47.4% female; median NIHSS on admission [IQR]: 7 (4, 14)), who received the standard of care. No statistically significant between-group differences were revealed in the PS-matched cohort, with the exception of a higher NIHSS on admission in the control group. The patients’ characteristics post-PS matching, stratified by melatonin treatment, are presented in Table 2.

### 3.2. Effects of Melatonin on Primary and Secondary Outcomes

In the full cohort, melatonin was not associated with mortality at discharge (ORadj: 0.462; 95% CI: 0.068–3.115; *p* = 0.427) after adjustment for age and prior DOAC treatments (hypercholesterolemia was not included in the model due to the lack of clinical evidence of an etiological association with post-ICH mortality). Similarly, in the adjusted logistic regression analyses, no associations were uncovered between melatonin and a good functional outcome at discharge (ORadj: 1.840; 95% CI: 0.776–4.361; *p* = 0.166), 90-day mortality (ORadj: 0.824; 95% CI: 0.282–2.407; *p* = 0.724) and a good functional outcome at 90 days (ORadj: 1.943; 95% CI: 0.914–4.131; *p* = 0.084).

**Figure 1:**
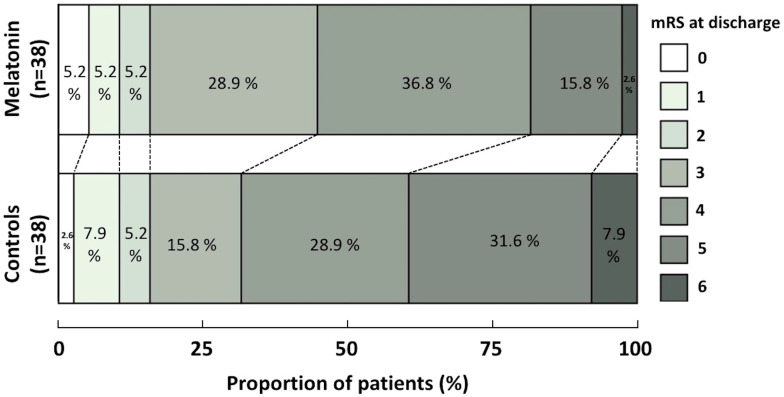
Ordinal mRS shift analysis at discharge. This analysis is based on the proportional odds (cumulative logit) model, adjusted for NIH Stroke Scale Score, in the propensity-score-matched cohort.

**Figure 2:**
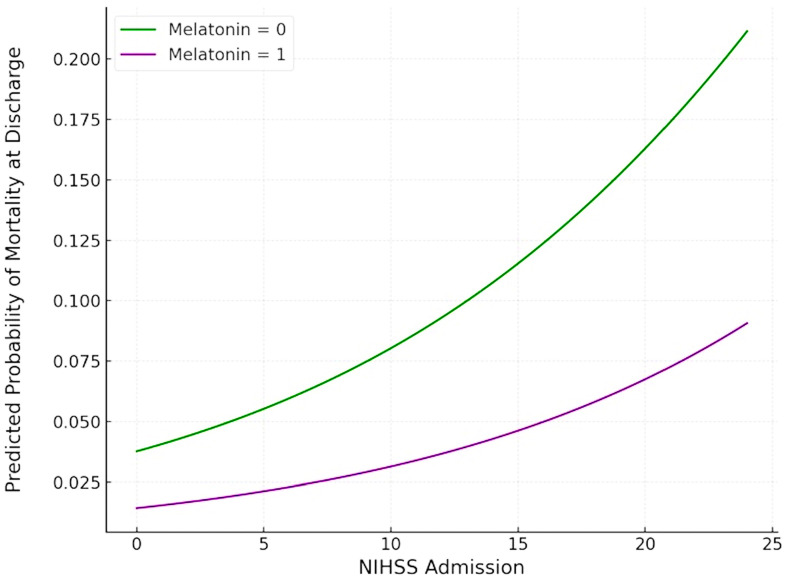
Plot of logistic regression analysis of mortality at discharge, adjusted for NIH Stroke Scale Score. Purple and green curves correspond to melatonin-treated patients and controls, respectively.

In the PS-matched cohort, mortality at discharge was three times lower in the melatonin-treated group compared to the control group (2.6% vs. 7.9%) (Figure 1). Nevertheless, the trend towards reduced mortality with melatonin did not reach statistical significance (ORadj: 0.372; 95% CI: 0.036–3.843; *p* = 0.407) (Figure 2). Similarly, although a higher NIHSS on admission was associated with a trend towards an increased risk of death at discharge, this association was non-significant (ORadj: 1.083; 95% CI: 0.922–1.273; *p* = 0.331).

**Figure 3:**
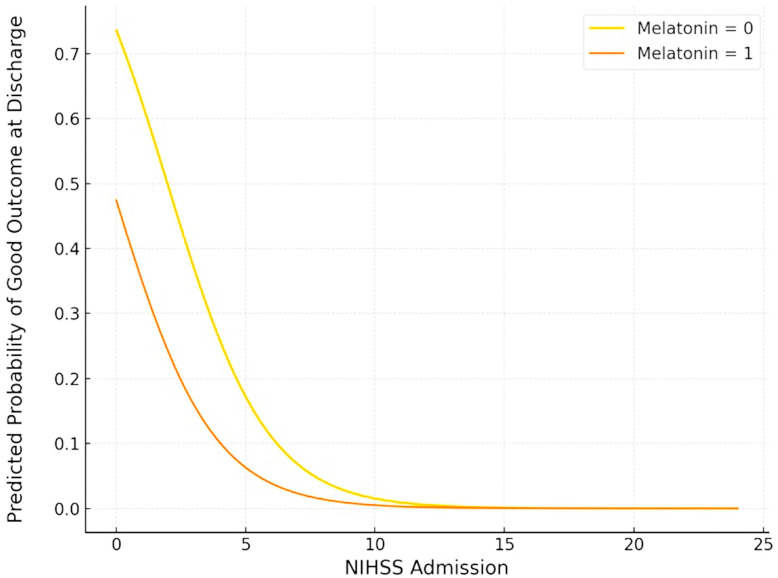
Plot of logistic regression analysis of favorable outcome (mRS ≤ 2) at discharge, adjusted for NIH Stroke Scale Score. Orange and yellow curves correspond to melatonin-treated patients and controls, respectively.

With respect to functional outcomes, severe disability (mRS = 5) at discharge was half as frequent in the melatonin-treated group compared to the control group (15.8% vs. 31.6%) (Figure 1). However, melatonin was not associated with the probability of a good functional outcome (ORadj: 0.323; 95% CI: 0.065–1.605; *p* = 0.167) (Figure 3). In contrast, a higher NIHSS on admission was significantly associated with a reduced likelihood of a good functional outcome at discharge (ORadj: 0.595; 95% CI: 0.420–0.842; *p* = 0.003).

**Figure 4:**
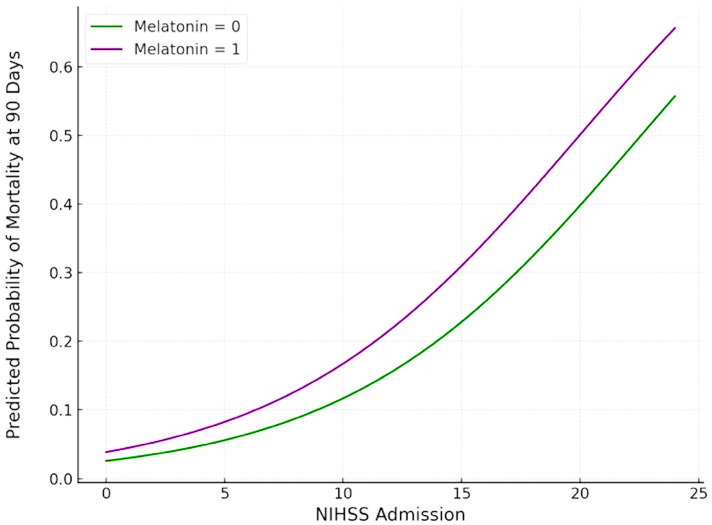
Plot of logistic regression analysis of mortality at 90 days post-ICH, adjusted for NIH Stroke Scale Score. Purple and green curves correspond to melatonin-treated patients and controls, respectively.

**Figure 5:**
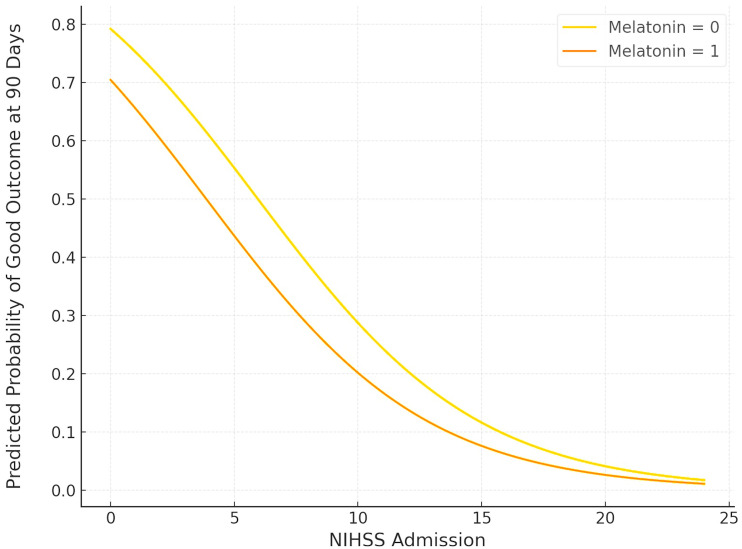
Plot of logistic regression analysis of favorable outcome (mRS ≤ 2) at 90 days post-ICH, adjusted for NIH Stroke Scale Score. Orange and yellow curves correspond to melatonin-treated patients and controls, respectively.

Similar results were observed for mortality and functional outcomes at the 90-day follow-up. In particular, no association was uncovered between the melatonin treatment and 90-day mortality (ORadj: 1.519; 95% CI: 0.295–7.826; *p* = 0.617) (Figure 4). In contrast, a higher NIHSS on admission was significantly associated with 90-day mortality (ORadj: 1.175; 95% CI: 1.029–1.340; *p* = 0.017). Regarding functional outcomes, melatonin was not associated with a higher probability of a good outcome at 90 days (ORadj: 0.626; 95% CI: 0.198–1.983; *p* = 0.426) (Figure 5). In contrast, a higher NIHSS on admission was significantly associated with lower odds of a good functional outcome at 90 days (ORadj: 0.799; 95% CI: 0.699–0.913; *p* < 0.001).

In the ordinal analysis of the mRS score at discharge, patients in the melatonin group had a median score of 4 (IQR: 3 to 4), which indicated a similar functional outcome to the median score of 4 (IQR: 3 to 5) among patients in the control group (common odds ratio, 0.762; 95% CI, 0.327 to 1.773; *p* = 0.527) (Figure 1).

In the full cohort (*n* = 177 patients), secondary logistic regression analyses—with a favorable outcome defined as an mRS of 0–3 and adjustment for age and prior DOAC treatment—revealed a significant association between melatonin and good functional outcomes at discharge (ORadj: 2.411; 95% CI: 1.217–4.776; *p* = 0.012), which was non-significant at 90 days (ORadj: 1.550; 95% CI: 0.766–3.136; *p* = 0.223). In the PS-matched cohort (*n* = 76 patients), after adjustment for NIHSS on admission, no associations were uncovered between melatonin and a good functional outcome at discharge (ORadj: 0.825; 95% CI: 0.239–2.845; *p* = 0.761) or at 90 days (ORadj: 0.639; 95% CI: 0.204–2.004; *p* = 0.442).

## 4. Discussion

The findings of this single-center, non-randomized, prospective cohort study comprising 177 consecutive patients with spontaneous ICH can be summarized as follows: First, in the primary analysis, melatonin was not associated with reduced mortality or improved functional outcomes at discharge or 90 days. Second, in the secondary analysis of a PS-matched cohort of 76 patients, mortality at discharge was three times lower in melatonin-treated patients compared to controls (2.6% vs. 7.9%); however, the trend toward reduced mortality did not reach statistical significance (ORadj: 0.372; 95% CI: 0.036–3.843; *p* = 0.407). Third, while severe disability (mRS = 5) at discharge was half as frequent in the melatonin-treated group compared to the control group (15.8% vs. 31.6%), this did not translate to a higher probability of achieving a good functional outcome (ORadj: 0.323; 95% CI: 0.065–1.605; *p* = 0.167). An excellent functional outcome (mRS = 0) at discharge was also numerically twice as frequent in the melatonin-treated group compared to the control group (5.25% vs. 2.6%), a small but potentially clinically meaningful difference. Even though statistical significance was not achieved, these findings suggest a potential benefit in terms of reducing disability that warrants further investigation. Fourth, our ordinal mRS shift analysis confirmed that the functional outcomes at discharge were comparable between melatonin-treated ICH patients and those receiving the standard of care (common odds ratio: 0.762; 95% CI: 0.327–1.773; *p* = 0.527). Fifth, there was no evidence of an association between the melatonin treatment and either mortality or functional outcome at 90 days post-ICH.

The present results require caution in their interpretation and should be considered within the context of certain methodological limitations. Notably, patients with an ICU/SU stay of less than 24 h, those requiring mechanical ventilation, and those who underwent palliative care or neurosurgical intervention immediately following their baseline CT scan were excluded from this analysis. It should be noted, however, that the exclusion of patients with extreme ICH severity (either very severe or minor cases) may have introduced selection bias, narrowing the dataset, reducing variability, and potentially increasing the risk of Type II errors by obscuring treatment effects. In particular, as no mechanically ventilated patients were treated at this study’s ICU/SU, data from patients under mechanical ventilation were not included in the present analysis. Since this may have introduced selection bias towards improved mortality, future well-designed trials including both ventilated and non-ventilated patients with ICH are warranted to robustly evaluate the potential effects of melatonin on mortality and functional outcomes.

In addition, the present analysis relies on data collected in the context of PSD prevention in stroke patients, following an SOP that was internally developed, validated, and implemented at this study’s SU [25,28]. This protocol included a daily dosage of 2 mg of melatonin for PSD prevention, a dosage that has previously been correlated with a significant reduction in the risk of PSD when administered within 24 h of acute ischemic stroke onset [28]. It is important to highlight, however, that much higher doses of melatonin have been utilized in prior research in ICH, including rodent experimental ICH models (ranging from 5 mg/kg to 50 mg/kg) [21,31] and a previous randomized, non-controlled, double-blind study in patients with non-traumatic ICH, which demonstrated a reduction in ICU stay duration with a standard daily dose of 30 mg [22]. Given that the melatonin dosage was likely subtherapeutic and that, due to melatonin’s short half-life, a 2 mg dose may have been insufficient for sustained neuroprotection, while also considering that RCTs and meta-analyses have consistently confirmed the tolerability and safety of high-dose melatonin (≥10 mg) in adults, the potential for missed dose-dependent effects on ICH mortality and functional outcomes warrants further investigation in well-designed future trials [22,32].

Some further aspects should be considered. First, a line of evidence suggests that circulating melatonin levels may correlate with neuronal injury and clinical outcomes following ICH [33]. In particular, elevated concentrations of endogenous melatonin in patients with non-traumatic ICH have been associated with reduced survival and increased levels of circulating malondialdehyde, a lipid peroxidation product and biomarker of oxidative stress [34]. These effects have been attributed to an “overshooting” response—namely, a counterbalancing upsurge in melatonin aimed at mitigating oxidative stress after ICH. Second, exogenous melatonin administration has been shown to replicate the antioxidant effects of its endogenous counterpart, including directly scavenging reactive oxygen and nitrogen species (ROS/RNS), activating antioxidative cascades, and targeting mitochondria [35]. In experimental ICH models, melatonin has also been associated with improved motor outcomes and the preservation of the corticospinal tract, along with the attenuation of hyperglycemia-induced brain injury via the PPARδ/PGC-1α pathway [36,37]. Third, due to its amphiphilic structure, melatonin can cross the blood–brain barrier and penetrate brain cells and their organelles, such as their mitochondria. This unique property enables melatonin to act as a potent non-enzymatic antioxidant both intra- and extracellularly, making it a highly attractive neuroprotective candidate for ICH [35,38]. Fourth, melatonin can improve sleep patterns, reduce delirium, and enhance cognitive recovery in critically ill patients [20,21]. Given the central role of sleep in neuroplasticity, cognitive function, and inflammation regulation—all of which impact post-ICH recovery—future trials should incorporate objective sleep assessments (e.g., actigraphy) and standardized procedures to optimize sleep and circadian rhythms (e.g., light therapy, structured sleep protocols). Such trials are warranted to discern whether melatonin’s primary mechanism of action in ICH is through direct neuroprotective effects or via improved sleep and sleep-mediated neuronal recovery.

Contrary to findings from preclinical studies, but in line with the lack of a statistically significant reduction in mortality observed in a previous non-randomized clinical study [22], the daily administration of 2 mg of melatonin showed no significant benefit in improving survival or functional outcomes post-ICH. Nevertheless, in our study, which included an almost fourfold larger sample of ICH patients [22], we observed a non-significant trend toward reduced mortality and severe disability among ICH patients, which warrants further investigation in the context of well-designed and adequately powered RCTs. As this trend was notable at discharge but vanished or even reversed at the 90-day follow-up post-ICH, several hypotheses could explain these findings, including the possibility of early anti-inflammatory or antioxidant effects that wane over time or that differences in post-discharge care or rehabilitation may account for the discordant findings observed at discharge and 90 days. Thus, future studies with longer follow-up periods should also aim to standardize ICH management in the post-discharge setting to enable meaningful comparisons between groups.

Some additional limitations of the current study include its monocentric, non-randomized design and the presence of potential biases and confounders not accounted for in the statistical analysis. Furthermore, it is important to acknowledge the limitations of the mRS as a measure of post-ICH disability, including its insensitivity to subtle changes in functional status and its inability to assess non-motor outcomes, such as cognitive impairment, which are relevant to stroke rehabilitation [39]. As a result, potential neurocognitive benefits of melatonin that were not explicitly evaluated in the present study may have been missed and should be addressed in future trial protocols [40,41,42,43].

## 5. Conclusions

In conclusion, melatonin supplementation in patients with spontaneous ICH demonstrated no efficacy in reducing mortality or improving their functional outcomes. Nonetheless, due to the methodological limitations of the present study, as outlined above, we cannot exclude the possibility that its therapeutic utility has been underestimated. The compelling preclinical data, combined with the well-established safety profile of melatonin and its cost-effectiveness, support clinical equipoise and the need for testing melatonin in the context of well-designed RCTs with appropriate dosing regimens in ICH patients.

## Figures and Tables

**Table 1:** Baseline characteristics of patients with ICH, based on whether they received melatonin treatment. The asterisk indicates statistical significance.

	All ICH Patients *n* = 177	ICH Patients Treated with Melatonin *n* = 84	ICH Patients Without Melatonin *n* = 93	*p*-Value
Age, y mean ± SD	71.4 ± 14.8	75.3 ± 12.8	67.8 ± 15.5	<0.001 *
Sex, female, *n* (%)	82 (46.3%)	38 (45.2%)	44 (47.3%)	0.076
Baseline clinical variables/scales on admission				
pmRS, median (IQR)	0 (0, 2)	0 (0, 2)	0 (0, 2)	0.501
NIHSS, median (IQR)	6 (3, 10)	5 (2, 9)	6 (3, 12)	0.084
GCS, median (IQR)	15 (14, 15)	15 (14, 15)	15 (12, 15)	0.126
ICH-score, median (IQR)	1 (0, 2)	1 (0, 2)	1 (0, 2)	0.831
Risk factors *n* (%)				
Arterial hypertension	151 (85.3%)	71 (84.5%)	80 (86%)	0.779
Diabetes mellitus	36 (20.3%)	13 (15.5%)	23 (24.7%)	0.127
Atrial fibrillation	45 (25.4%)	24 (28.6%)	21 (22.6%)	0.361
Hypercholesterolemia	36 (20.3%)	23 (27.4%)	13 (14%)	0.027 *
Smoking	27 (15.3%)	9 (10.7%)	18 (19.4%)	0.110
Alcohol	16 (9%)	8 (9.5%)	8 (8.6%)	0.831
Obesity (BMI > 30)	36 (20.3%)	16 (19%)	20 (21.5%)	0.685
Coronary heart disease	28 (15.8%)	17 (20.2%)	11 (11.8%)	0.126
Malignancy	9 (5.1%)	6 (7.1%)	3 (3.2%)	0.236
Dementia	19 (10.7%)	12 (14.3%)	7 (7.5%)	0.147
Anticoagulation on admission, *n* (%)	39 (22%)	23 (27.4%)	16 (17.2%)	0.103
Vitamin-K antagonist	15 (8.5%)	7 (8.3%)	8 (8.6%)	0.949
Direct oral anticoagulants	24 (13.6%)	16 (19%)	8 (8.6%)	0.043 *
Antithrombotic therapy on admission, *n* (%)				
Single	39 (22%)	18 (21.4%)	21 (22.6%)	0.853
Dual	6 (3.4%)	3 (3.6%)	3 (3.2%)	0.899
Imaging, *n* (%)				
Supratentorial localization				
Deep	90 (50.8%)	39 (46.4%)	51 (54.8%)	0.264
Lobar	69 (39.0%)	35 (41.7%)	34 (36.6%)	0.487
Infratentorial localization				
Brainstem	7 (4%)	2 (2.4%)	5 (5.4%)	0.307
Cerebellum	15 (8.5%)	9 (10.7%)	6 (6.5%)	0.309
Siderosis	16 (9%)	9 (10.7%)	7 (7.5%)	0.460
IVH on admission	2 (1.1%)	0 (0%)	2 (2.2%)	0.176
FAZEKAS PWM median (IQR)	1 (0, 2)	1 (0, 2)	1 (0, 2)	0.198
FAZEKAS DWM median (IQR)	1 (0, 3)	2 (0, 3)	2 (0, 3)	0.172
ICH volume [cm^3^], median (IQR) on admission	9 (4, 19)	8 (3, 16)	9 (4, 23)	0.390
Etiology SMASH-U, *n* (%)				0.197
Structural vascular lesion	8 (4.5%)	3 (3.6%)	5 (5.4%)	
Systemic/other disease	10 (5.6%)	5 (6%)	5 (5.4%)	
Medication	34 (19.2%)	22 (26.2%)	12 (12.9%)	
Amyloid angiopathy	23 (13%)	13 (15.5%)	10 (10.8%)	
Hypertension	79 (44.6%)	32 (38.1%)	47 (50.5%)	
Undetermined	23 (13%)	9 (10.7%)	14 (15.1%)	

**Table 2:** Baseline characteristics of patients with ICH after propensity score matching, based on whether they received melatonin treatment. The asterisk indicates statistical significance.

	All ICH Patients *n* = 76	ICH Patients Treated with Melatonin *n* = 38	ICH Patients Without Melatonin *n* = 38	*p*-Value	SMD
Age, y mean ± SD	76.1 ± 10.9	76.2 ± 11.1	75.9 ± 10.9	0.876	0.017
Sex, female, *n* (%)	34 (44.7%)	16 (42.1%)	18 (47.4%)	0.645	
Baseline clinical variables/scales on admission					
pmRS, median (IQR)	0 (0, 2)	0 (0, 2)	0 (0, 2)	0.501	
NIHSS, median (IQR)	6 (3, 11)	5 (1, 9)	7 (4, 14)	0.024 *	
GCS, median (IQR)	15 (13, 15)	15 (14, 15)	14 (11, 15)	0.156	
ICH-score, median (IQR)	1 (0, 2)	1 (0, 2)	1 (1, 2)	0.586	
Risk factors *n* (%)					
Arterial hypertension	68 (89.5%)	33 (86.8%)	35 (92.1%)	0.455	
Diabetes mellitus	13 (17.1%)	5 (13.2%)	8 (21.1%)	0.361	
Atrial fibrillation	18 (23.7%)	9 (23.7%)	9 (23.7%)	1.000	
Hypercholesterolemia	11 (14.5%)	5 (13.2%)	6 (15.8%)	0.744	0.037
Smoking	9 (11.8%)	4 (10.5%)	5 (13.2%)	0.723	
Alcohol	8 (10.5%)	5 (13.2%)	3 (7.9%)	0.455	
Obesity (BMI > 30)	13 (17.1%)	4 (10.4%)	9 (23.7%)	0.128	
Coronary heart disease	11 (14.5%)	6 (15.8%)	5 (13.2%)	0.744	
Malignancy	6 (7.9%)	3 (7.9%)	3 (7.9%)	1.000	
Dementia	7 (9.2%)	4 (10.5%)	3 (7.9%)	0.692	
Anticoagulation on admission, *n* (%)	16 (21.1%)	10 (26.3%)	6 (15.8%)	0.260	
Vitamin-K antagonist	9 (11.8%)	6 (15.8%)	3 (7.9%)	0.287	
Direct oral anticoagulants	7 (9.2%)	4 (10.5%)	3 (7.9%)	0.692	0.046
Antithrombotic therapy on admission, *n* (%)					
Single	17 (22.4%)	8 (21.1%)	9 (23.7%)	0.783	
Dual	4 (5.3%)	2 (5.3%)	2 (5.3%)	1.000	
Imaging, *n* (%)					
Supratentorial localization					
Deep	35 (46.1%)	14 (36.8%)	21 (55.3%)	0.107	
Lobar	37 (48.7%)	20 (52.6%)	17 (44.7%)	0.491	
Infratentorial localization					
Brainstem	0 (0%)	0 (0%)	0 (0%)	-	
Cerebellum	6 (7.9%)	4 (10.5%)	2 (5.3%)	0.395	
Siderosis	10 (13.2%)	5 (13.2%)	5 (13.2%)	1.000	
IVH on admission	0 (0%)	0 (0%)	0 (0%)	-	
FAZEKAS PWM median (IQR)	1 (0, 2)	1 (1, 2)	1 (0, 2)	0.519	
FAZEKAS DWM median (IQR)	2 (0, 3)	2 (1, 3)	2 (0, 3)	0.759	
ICH volume [cm^3^], median (IQR) on admission	9 (4, 19)	9 (4, 18)	10 (4, 21)	0.803	
Etiology SMASH-U, *n* (%)				0.178	
Structural vascular lesion	0 (0%)	0 (0%)	0 (0%)		
Systemic/other disease	1 (1.3%)	1 (2.6%)	0 (0%)		
Medication	12 (15.8%)	9 (23.7%)	3 (7.9%)		
Amyloid angiopathy	16 (21.1%)	9 (23.7%)	7 (18.4%)		
Hypertension	35 (46.1%)	15 (39.5%)	20 (52.6%)		
Undetermined	12 (15.8%)	4 (10.5%)	8 (21.1%)		

## Data Availability

The raw data supporting the conclusions of this article will be made available by the authors upon reasonable request.
